# A comparative study of postgraduate theses in pedagogy and preschool education in Austria and Turkey

**DOI:** 10.3389/fpsyg.2022.1051923

**Published:** 2023-01-06

**Authors:** Yunus Pınar, Dilek Gür, Nihal Kubilay Pınar, Kemal Demir, Ekin Kaynak Iltar, Sevgi Arkılıç Songören, Salih Özenici

**Affiliations:** Department of Preschool Education, Akdeniz University, Antalya, Turkey

**Keywords:** preschool education, postgraduate theses, originality, quality of academic writing, comparative educational research, Austria and Turkey

## Abstract

In this study, postgraduate theses in the fields of pedagogy and preschool education in Austria and Turkey are compared in terms of factors such as similarity index, research designs and quality of academic writing. To achieve this goal, a commonly-used web-based plagiarism detection service was used to determine the similarity indexes of 258 theses prepared in the two countries (124 from Austria, 134 from Turkey) by checking them against existing sources such as articles, theses, and books; and a relational screening model was used to determine the degree of correlation among variables. In addition, the research topics, research designs, and data collection tools employed in each thesis were identified and content analysis was performed on selected theses with an eye to gaining a sense of the methodological approaches used in pedagogy in Austrian and Turkish universities and making comparisons between the two countries. Our results suggest that the mean similarity index between the postgraduate theses in Austria is 8.78 (Std. Dev. 4.91), while the mean similarity index between the postgraduate theses prepared in Turkey is 25.10 (Std. Dev. 9.85). Our analysis indeed indicates that 91% (*n* 113) of the theses prepared at Austrian universities and only 13% (*n* 17) of the theses prepared at Turkish universities did not exceed the acceptable similarity index of 15%. The fact that 87% of the theses written in Turkey are dramatically similar to the available resources shows that many of the studies carry potential risks in terms of originality and plagiarism.

## Introduction

Development of early childhood and the formal training of preschool teachers in Austria dates back 1872, when a law regulated this training for the first time (Smidt and Embacher, 2020). Since the 1870s, associations of schoolteachers pressured local school authorities to adopt kindergarten classes into public-school systems and in 1870 the Austrian teachers’ assembly set up a ‘kindergarten’ section (Willekens and Scheiwe, 2018). A reforming impetus after World War I brought about innovative pedagogical approaches and perspectives. Roubiczek developed a concept based on the findings of Montessori model and fused it with ideas in psychoanalysis, influenced by Viennese psychoanalysts such as Anna Freud und Erik Erikson. The first Montessori school (Children’s House) in Vienna in 1921/1922 was established by Maria Montessori based on psychoanalytical pedagogical ideas, in cooperation with Anna Freud who had founded a crèche in the same building (Willekens and Scheiwe, 2018).

Austria’s conservative childcare policy took an interesting turn in 2009, and ever since that date, the final preschool year has been obligatory for all children (Bauer and Mitterer, 2014; Smidt and Embacher, 2020). In Austria, early childhood teachers are trained at Training Institutes for Early Childhood Education (BAfEP), a vocational secondary school and, since 2021, also at teacher training colleges in the higher education program elementary pedagogy (Methlagl et al., 2022).

Nodoubtly, Austria can be viewed as one of the representative of modern economies and societies and places high importance on early education. As has been previously reported in the literature, after a first peak (which was initiated by the Sputnik crisis) especially in the late 1960s and early 1970s, early childhood education plays a crucial role in the educational system in Austria (Koch and Farquhar, 2015; Smidt, 2018). Preschools in Austria are funded by private and public sponsors and have attendance rates of around 93% for children aged 3–5 years old.

Despite being a small country, Austria has 22 state universities, 21 universities of applied sciences, 14 teacher training university colleges and 16 private universities. Compared with the situation in other countries, such as the United States or the United Kingdom, “*there is little prestige-based segregation among the state universities in Austria. The Austrian university landscape is therefore marked by low institutional differentiation”* (Lessky et al., 2022). It is important to note that Higher education institutions in Austria can be categorized as integrated systems as the academic staff take on tasks in both research and teaching. The professional contexts in higher education institutions in Austria and Germany share many similarities (Hein et al., 2021) and the academics in Austria spend most hours (39% of the working hours) on research-related activities (Kwiek and Antonowicz, 2013).

Unlike Austria, early childhood education (ECE) had been largely side lined in the first decades of the new Turkish Republic (1923), mostly as a consequence of the prioritization of primary education (Bekman, 2005) and is not deeply rooted in the history of Turkish education and tradition. Although there were some institutions providing a kind of education for young children during the Ottoman Empire Period (Ural and Ramazan, 2007; Altun et al., 2011) and although the Ottoman elementary schools were known to be the first ECE schools, the first schools in the modern sense were opened in the early 1910s by the Party of Union and Progress (Toran, 2012). According to Bekman (2005) Turkey does not have standardized widespread system of ECE programs and after the 1990’s ECE began to receive the attention it deserved and a large number of studies and programs have been conducted since then. From the academic year 1991–1992 onwards, departments of early childhood education (or: preschool education) were opened at the faculties of education in some Turkish universities (Oktay, 1999). Over the ensuing years, it was tried to train teachers for the institutions of preschool education with the educational programs opened at higher education level (Kandır and Yazıcı, 2016). It should be pointed out in the context of the training of preschool teachers that graduates from Girls’ Vocational High School and from various education faculties’ two-year pre-license programs could become preschool teachers (Kapci and Guler, 1999). After 1991, in order to become a preschool teacher one had to graduate from the education faculty’s preschool teacher department. Until 1998, each faculty could decide their own program, which caused differences in teacher quality. Only after 1998–1999, all the education faculties have started to apply the same framed curriculum for teacher preparation (Kapci and Guler, 1999).

As a country type, *Turkey can be considered a middle-size power broadly defined as a state that is able to exert influence on a regional and, to some degree, global scale* (Aydinli and Mathews, 2021). A review of the literature revealed that the history of modern higher education in Turkey goes back to 19th-century trials with some struggles of modernization it takes the form of a modern university mostly after the establishment of the modern and Westernized Turkish Republic in 1923 (Kalkan, 2019). Over approximately the last 15 years, the higher education in Turkey changed from that of a “selective elitist institution to mass higher education” (Cin et al., 2021). It can be concluded that the most dramatic increase in the number of public universities came after 2005. The policy proposed that every province across the country would have at least one public university (Özoǧlu et al., 2016). This massive expansion was probably driven by the opening of “one public university in each city” that did not already have one, which led to the establishment of 41 new public universities between 2006 and 2008 alone. As a consequence of the massive expansion the total number of Turkish universities has reached 208, of which 129 states, 75 are foundation and 4 are foundation vocational schools (Higher Education Information Management System, 2022). The majority of these universities are located in economically vibrant cities and attract students from diverse backgrounds (Cin et al., 2021). It should be underlined that, although the number of faculty members working in higher education institutions has increased continuously over the years, the number of students per academics is still quite high (Gür et al., 2017).

## Plagiarism of academic writing

It can be assumed that one of the objective ways to determine the frequency of plagiarism is by using text-matching software, which detects textual similarity between the submitted paper and a large database of scientific books, papers and webpages etc. (Pupovac, 2021). Several studies in the literature have been conducted to compare all (in some cases more than 750,000) papers from a particular bibliographic database against each other to identify similarity indexes and plagiarism (Errami et al., 2008; Sun et al., 2010; Citron and Ginsparg, 2015). Interestingly, Pupovac (2021) states that “*No human is able to analyze such a large sample of articles and compare them against such comprehensive databases of relevant scientific literature as is possible with software.”* It should be emphasized, however, that text-matching softwares do not identify plagiarism, only textual similarity between papers; therefore, human interpretation of findings is necessary to acquire more trustworthy results (Pupovac, 2021). On the other hand, it should be noted that the studies, using text-matching software to determine the similarity indexes can provide important clues on academic writing quality or possible plagiarism.

The literature review showed that little attention has been paid to the similarity index percentage of master’s and doctoral theses written in preschool education departments in Austria and Turkey, the frequency of possible plagiarism, such as the investigation of methodological approaches of these countries in this field. A review of previous literature revealed that there is a high degree of awareness about plagiarism in Austria. An empirical survey with 543 students which completed a survey representing 15 institutions in Austria demonstrated that students were particularly knowledgeable about what could be done to reduce plagiarism, their suggestions and those from teacher respondents should be studied by those people in Austria that are currently working on policies (Michałowska-Dutkiewicz et al., 2013).

Toprak has carried out a study by using text-matching software on how frequently plagiarism is observed in postgraduate theses in Turkey, to raise awareness of some aspects of plagiarism. The findings of this study suggest that “*the Turkish academic writing at the graduate level is alarming and worrisome in relation to quality and ethics of academic writing”* (Toprak and Yücel, 2020). In this retrospective study, which examines 600 graduate theses has been found that 34.5% the examined theses include plagiarism and the originality of theses has been found to be 28.7%.

## Objectives and questions

Comparative studies arguably have an important place in many disciplines such as educational sciences, politics, anthropology, sociology, history, medicine etc. (de Albuquerque Moreira et al., 2019). Comparative studies are essential in the investigation of the efficacy of drugs or treatment methods. Similarly, assessment methods like PISA may be required to evaluate the performance of the educational systems of different countries.

In this study, postgraduate theses in the fields of pedagogy and preschool education at universities in two OECD countries, Austria and Turkey, were comparatively analyzed using a set of variables. We believe that results informed by a comparative analysis of postgraduate theses prepared in these two countries, which are quite different in terms of their populations and socio-cultural characteristics, will be of interest to researchers from various disciplines, primarily educational sciences.

The objective of this study is to get a sense of the quality of academic writing in Austria and Turkey by comparing the originality and similarity indexes of master’s and doctoral theses produced in the two countries on subjects related to early childhood. In addition, the distribution of the theses based on their research designs as well as the paradigms and perspectives adopted by investigators in early childhood studies in the two countries will be outlined.

## Research questions

The following questions were investigated within the scope of the study:

1. What methodological approaches are used in pedagogy at Austrian and Turkish universities? Do the scientific perspectives of the two countries differ from each other?
2. What is the mean similarity index percentage of master’s and doctoral theses written in preschool education departments in Turkey?
3. What is the mean similarity index percentage of master’s and doctoral theses written in the field of pedagogy (for early childhood) in Austria?
4. Do the similarity indexes of postgraduate theses written in preschool education departments in Turkey vary significantly over years?
5. Do the similarity indexes of postgraduate theses written in the field of pedagogy in Austria vary significantly over years?
6. Is there a significant difference between the similarity index percentage of master’s and doctoral theses in preschool education in Turkey and in pedagogy in Austria?
7. Do the similarity indexes of postgraduate theses in preschool education departments in Turkey vary significantly across different universities? If there a variation exists, which three Turkish universities produce the most original theses?
8. Do the similarity indexes of postgraduate theses in pedagogy in Austria vary significantly across different universities? If a variation exists, which three universities produce the most original theses?
9. Do the similarity indexes of master’s and doctoral theses in Turkey vary significantly?
10. Do the similarity indexes of master’s and doctoral theses in Austria vary significantly?

## Methods and data collection tools

In this study, a relational screening model and content analysis were used. Relational screening models are research tools aimed at identifying the presence and/or degree of concurrent change between two or more variables (Christensen et al., 2011). In this paper, we compared and evaluated 258 selected master’s and doctoral theses in the field of preschool education in Austria and Turkey using the Turnitin application and by employing statistical analysis techniques.

The web-based service Turnitin, which is used to prevent plagiarism and to detect content that is not original, was used in this study. Established in 1997, Turnitin is a US-based company that specializes in the detection of plagiarism and the creation of similarity reports; its services are used by numerous institutions and organizations (Ireland, Christopher, and English, John, 2011). The Turnitin database has access to over 60 billion websites and more than 600 million student assignments. Academic work uploaded to the Turnitin website is compared to existing materials accessed by this database to produce a similarity index for the uploaded work. Although a high similarity index does not necessarily mean that the uploaded work contains plagiarism, it is recommended that materials with similarity index above 15% should be carefully reviewed. Obviously, an academic work with a similarity index of more than 15% cannot be said to be plagiarized with certainly. Conversely, one cannot claim with certainty that a work with a similarity index below 15% is plagiarism-free. A detailed investigation needs to be carried out to identify the presence of any plagiarism in any academic work. Nevertheless, a similarity index of 15% is a critical level. It is generally assumed that materials with similarity indexes above this level do not fully comply with the rules of academic writing.

The theses selected in this study were classified according to codes and categories prepared by the investigators, and the topics and research designs of the theses and the data collection tools used therein were evaluated through content analysis (Bowen, 2009).

## Sampling

**Figure 1:**
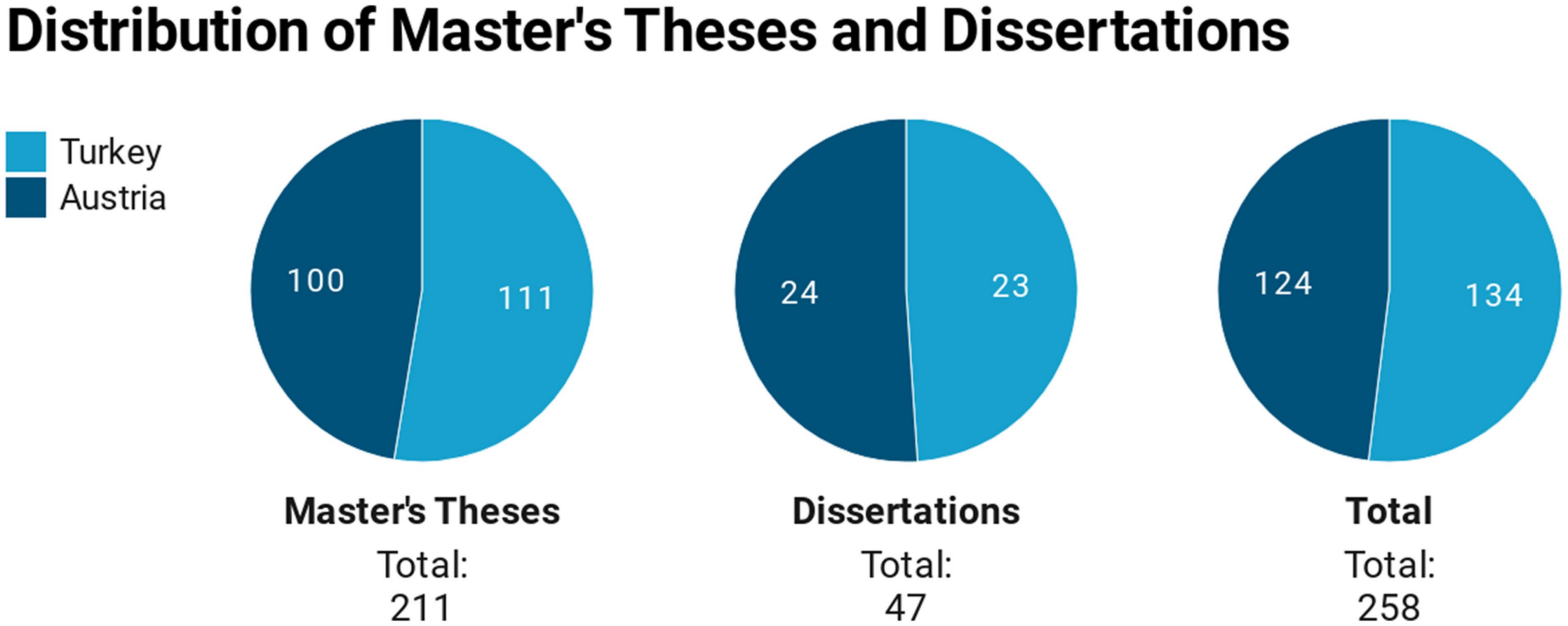
Distribution of theses by country.

Within the scope of the study, 134 postgraduate theses randomly selected from the master’s and doctoral theses written at preschool education departments in Turkey and that were accessible through the National Thesis Center of the Publications and Documentation Department of the Higher Education Council (YÖK, 2019) were reviewed. In Austria, there is no national repository for postgraduate theses; only postgraduate theses that are accessible *via* university libraries are available. Thus, 124 postgraduate theses selected randomly from the master’s and doctoral theses in the field of pedagogy at universities that grant access were reviewed. The distribution of these theses by country and type of thesis is shown in Figure 1.

As seen in Figure 1, 111 of the 134 theses that make up the sample in Turkey are master’s theses and 23 are doctoral theses. In the same manner, 100 of the 124 postgraduate theses that make up the Austrian sample are master’s theses and 24 are doctoral theses.

**Figure 2:**
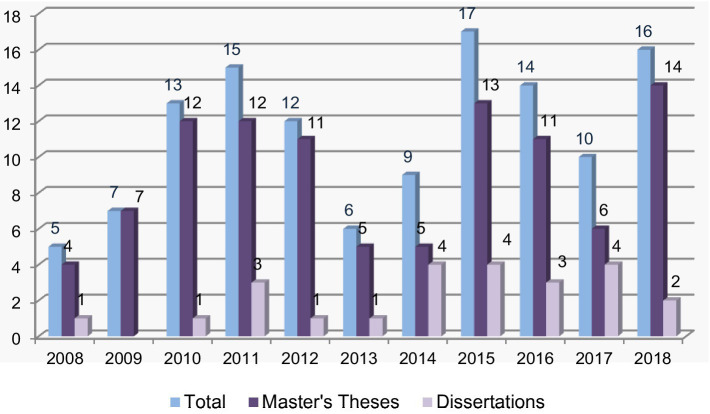
Distribution of master’s theses and PhD dissertations of the Austrian sample by year.

**Figure 3:**
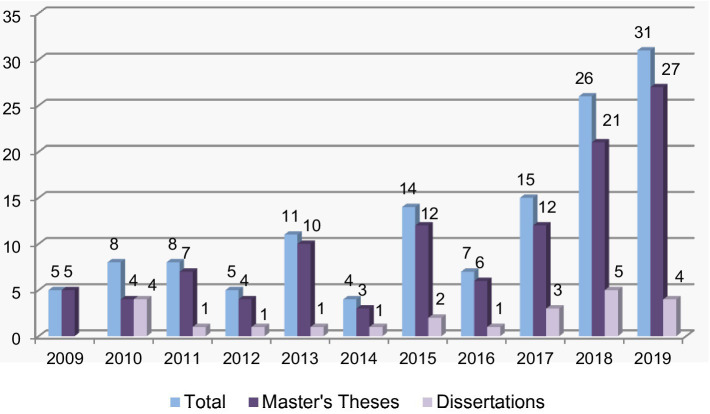
Distribution of master’s theses and PhD dissertations of the Turkish sample by year.

Figures 2, 3 show, respectively, the distributions of the postgraduate theses comprising the Austrian and Turkish samples by year.

As seen in Figure 2, in the Austrian sample, the largest number of postgraduate theses (17) were written in 2015 and the smallest number (5) were written in 2008. Also, the largest number of master’s theses (16) were written in 2018, and a higher number of doctoral theses were written in 2014, 2015 and 2017 compared to other years.

In comparison, as seen in Figure 3, in the Turkish sample, the largest number of postgraduate theses (31) were written in 2019 and the smallest number (4) were written in 2014. Notably, the largest number of master’s and doctoral theses were written in 2019.

## Data analysis and procedure

The theses included in the scope of the study using simple random sampling were first uploaded to the Turnitin website. Filters were applied in Turnitin settings to exclude the bibliography sections of the theses and matches with less than 5 words from the calculation of similarity indexes. Each reviewed thesis was encoded to protect their confidentiality and deleted from the database after the review was completed. The review process lasted approximately 8 months. Considering that pages such as the thesis cover page and the page with institutional approvals are standard and that every reviewed thesis would exhibit normal similarities to other works, similarity indexes arising from these were not included in the calculation. In the event an article was published in a journal or a conference paper was published in the proceedings of a conference based on a reviewed thesis and if these articles exhibited similarities to the author’s postgraduate thesis, these were not treated as self-plagiarism, and the similarity indexes arising from them were not included in the calculation.

If a reviewed thesis was indexed in university library systems (such as OpenMETU) and/or was published in full or in part on such systems, any similarity indexes arising from these were excluded from the calculation. Similarity indexes arising from direct quotes exceeding three lines, were excluded if the quote was in compliance with scientific research techniques. Overlaps caused by the presence of assessment instruments included in the appendices of numerous postgraduate theses and the resulting similarity indexes were also excluded.

Prior to the analysis of the data, the structure of the data was inspected to determine whether the assumptions related to the analyses to be performed were met. The Kolmogorov–Smirnov test performed to find out whether the entire data for Turkey and Austria and the data for each country exhibited a normal distribution showed that the data set did not exhibit a normal distribution (*p* = 0.000). However, an analysis of the skewness and kurtosis coefficients and the histograms for the data sets pointed at the potential existence of parallel results. Accordingly, the non-parametric Mann–Whitney U and Kruskal Wallis H tests were employed in the study.

To perform the content analyses, to improve the validity (communicative validity) of the analyses and to prevent potential errors, the investigators formed two separate study groups (Kvale, 1995). The first group comprised researchers who completed their doctoral studies in German-speaking countries; the second group consisted of researchers with a doctorate whose native language is Turkish. During “Content Analysis Seminar Meetings,” held once every 2 weeks, the researchers evaluated the theses both individually and collectively in line with the codes and categories that were created earlier.

## Findings

### Findings related to research question 1

We found that 94% or 116 of the 124 postgraduate theses written in Austria were qualitative-design studies. These theses employed a broad spectrum of qualitative research designs and data collection tools, ranging from observations according to Tavistock model (Datler et al., 2014), videography, video analysis (Reichertz and Englert, 2011), problem-centered interviews, narration interviews, content analysis, ethnography (Hammersley, 1992), Hermeneutics (Bereswill et al., 2010), Grounded Theory (Strauss and Corbin, 1998), biographical methods, and single and multiple case studies (George and Bennett, 2005).

**Figure 4:**
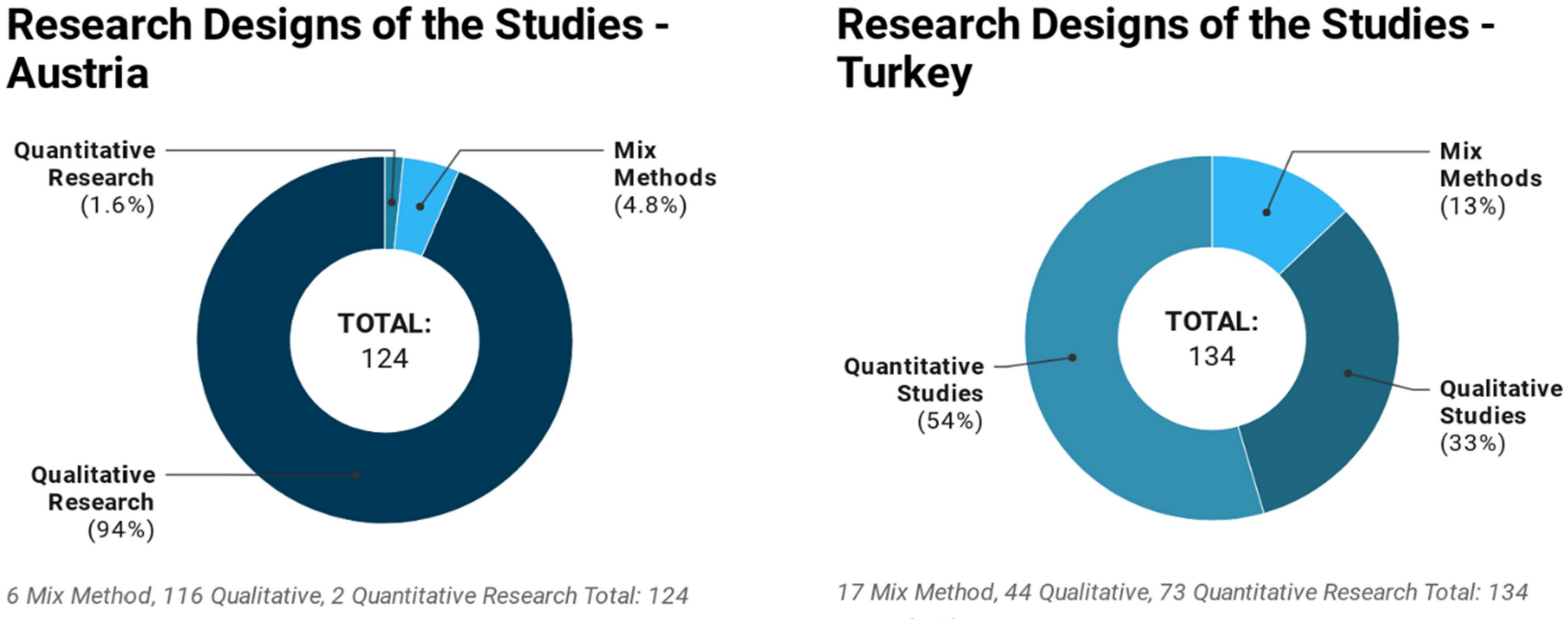
Distribution of master’s and PhD theses from Austria and Turkey by research design.

In comparison, 54% or 73 of the postgraduate theses written in Turkey used quantitative, 33% (*n* = 44) used qualitative, and 13% (*n* = 7) used mixed research methods such as that shown in Figure 4. Most of the quantitative studies were based on the use of relational screening, surveys, or assessment instruments, and some consisted of semi-experimental studies.

We found that 25% (*n* = 31) of the 124 postgraduate theses written in Austria were child-focused studies. Qualitative video analyses of kindergarten and preschool children, child observations and face-to-face interviews were dominant among these studies.^1^ Pictures drawn by children were analyzed in a small number of the theses. Further, 33% of the postgraduate theses in the Austrian sample were found to be adult-centered studies. These studies were mostly carried out using qualitative data collection methods with parents, mothers, fathers, preschool teachers, child therapists and school administrators. The remaining 42% of the postgraduate theses written in Austria were found to comprise other research topics. This group included theses focused on topics such as bilingualism, language acquisition, and institutions such as children’s museums, kindergartens, and nurseries as well as various documents, learning theories, learning plans and methods. In addition, a broad spectrum of studies on topics ranging from screen based media and video games to computer games, tablets and children’s books were noted.

**Figure 5:**
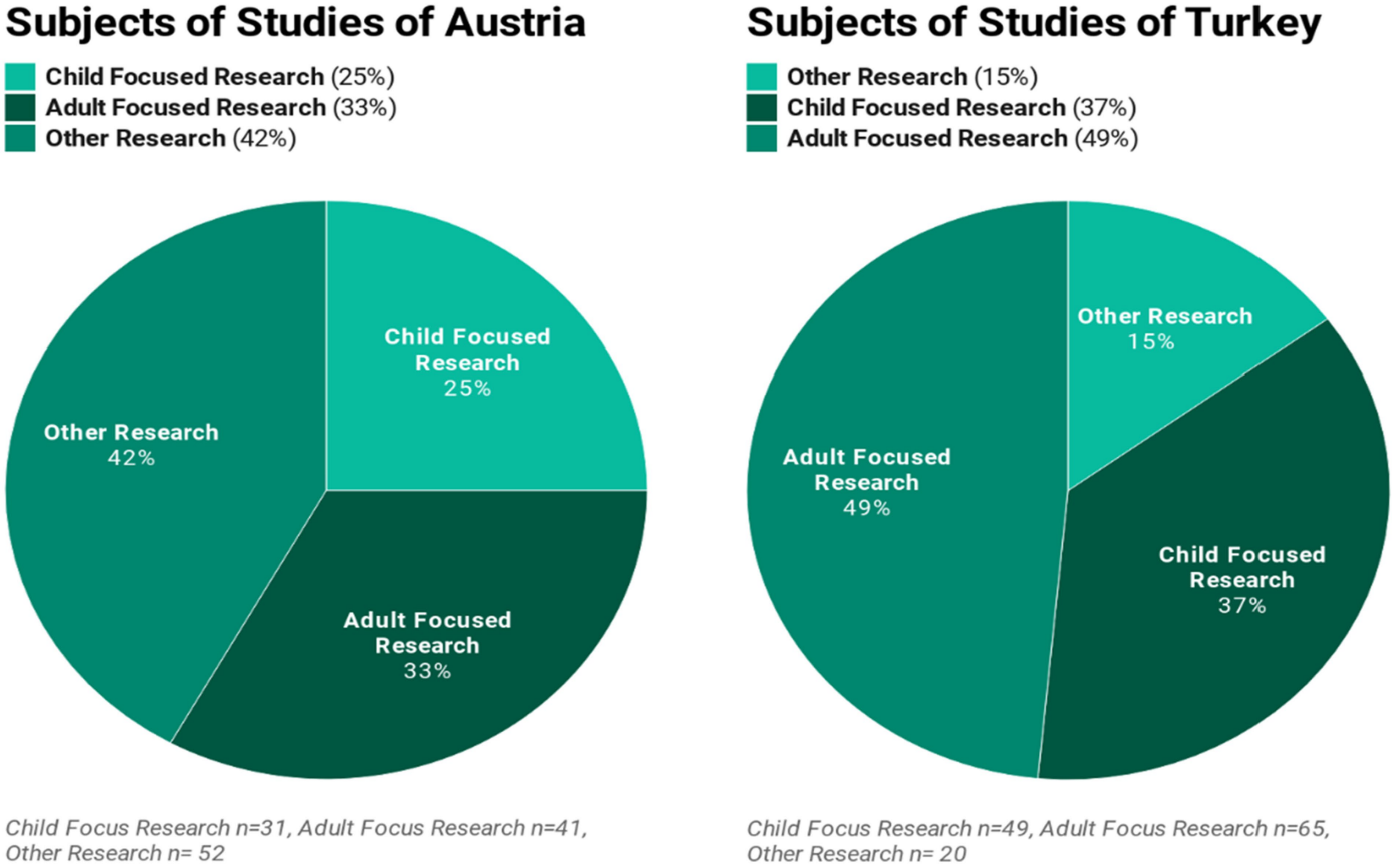
Distribution of research topics by country.

Most of the quantitative and qualitative studies conducted in Turkey were found to be aimed at preschool teachers, prospective preschool teachers or parents. Virtually none of the theses reviewed involved single-or multi-subject in-depth research about preschool children or was based on video analysis or ethnographic and psychoanalytic observations. We found that 49% of the postgraduate theses written in Turkey were adult-oriented studies that predominantly examined the views and attitudes of preschool teachers, university students who plan to be teachers and parents on various subjects.^2^ In studies where the pre-test/final-test model with experiments and control groups was used, mainly Family Information Forms, Teacher Behavioral Checklists, Family Behavioral Checklists and a variety of surveys and assessment instruments were used as data collection tools. Figure 5 shows the distribution of research topics among the two countries.

The child-focused theses in Turkey were found to be dominated by a research approach (or tradition) where the use of quantitative data collection tools is more common. Some of the research topics were discussed with the help of mostly outdated assessment instruments such as The Denver Developmental Screening Test (Frankenburg and Dodds, 1967), Preschool Behavior Questionnaire (Behar, 1977), Denham’s Affect Knowledge Test (Denham, 1986), Peabody Picture Vocabulary Test (Dunn and Dunn, 1965). Additionally, 15% of the postgraduate theses written in Turkey were found to comprise other research topics, such as children’s books, stories, language development, stage plays, educational programs and the physical structure of institutions such as kindergartens.

### Findings related to research questions 2 and 3

The mean and standard deviation values of similarity indexes of master’s and doctoral theses written at the preschool education departments in Turkey are presented in Table 1.

**Table 1:** Descriptive statistics for master’s and doctoral theses in Turkey.

Turkey	*n*	$\bar{X}$	SD
Master’s	111	26.63	9.83
Doctoral	23	17.74	5.98
Total	134	25.10	9.85

**Figure 6:**
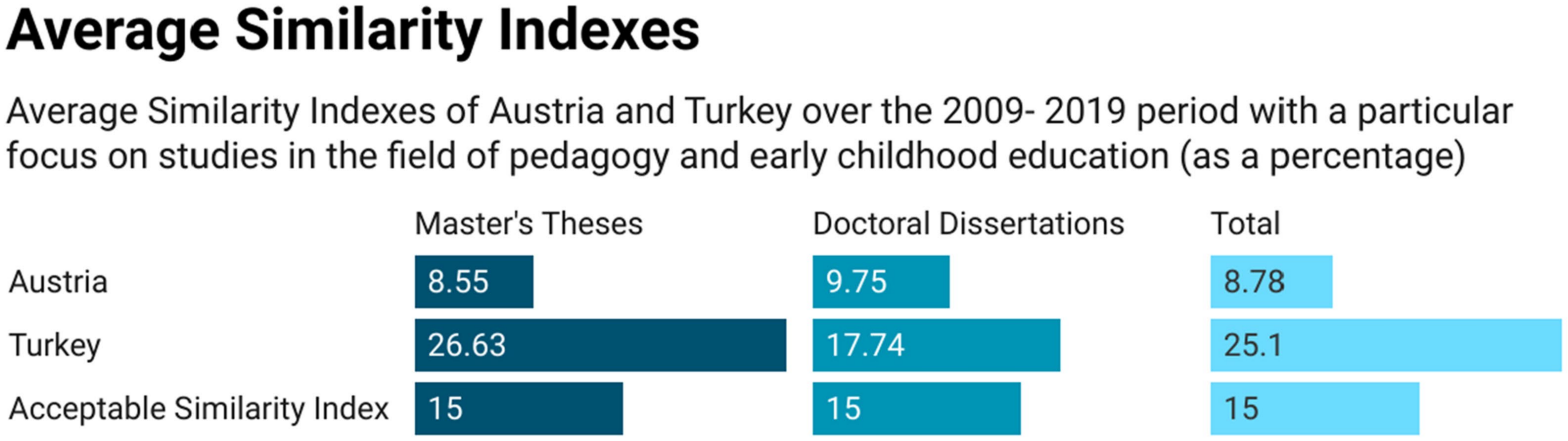
Average similarity indexes of Austria and Turkey.

As seen in Table 1, the mean similarity indexes of master’s theses written in Turkey was 26.63 with a standard deviation of 9.83; and the mean similarity index of doctoral theses was 17.74 with a standard deviation of 5.98. The mean similarity index of all postgraduate theses in Turkey was 25.10 with a standard deviation of 9.85 as shown in the Figure 6.

The mean and standard deviation values of similarity indexes master’s and doctoral theses written in Austria in the field of pedagogy (early childhood), are presented in Table 2.

**Table 2:** Descriptive statistics for master’s and doctoral theses in Austria.

Austria	*n*	$\bar{X}$	SD
Master’s	100	8.55	4.82
Doctoral	24	9.75	5.28
Total	124	8.78	4.91

**Figure 7:**
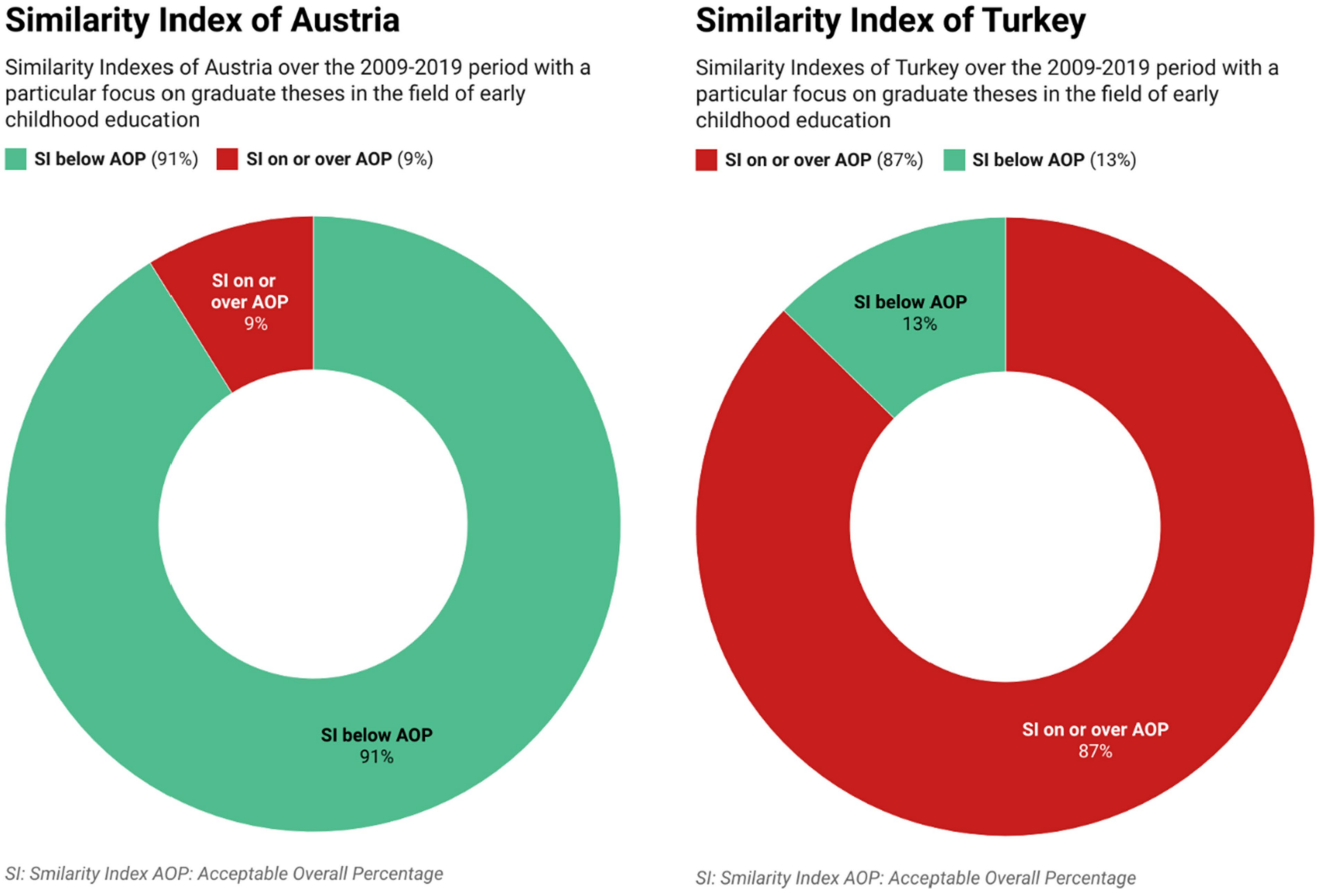
Similarity Indexes of Turkey and Austria.

As seen in Table 2, the mean similarity index of master’s theses written in Austria was 8.55 with a standard deviation of 4.82. Similarly, the mean similarity index of doctoral theses was 9.75 with a standard deviation of 5.28. The mean similarity index of all postgraduate theses in Austria was 8.78 with a standard deviation of 4.91. We found that 91% (*n* = 113) of the theses from Austria did not exceed the 15 percent similarity index which we considered as an acceptable overall percentage, and only 9% (*n* = 11) exceeded the critical level. In comparison, only 13% (*n* = 17) of the theses written in Turkey fell below the critical level while 87% (*n* = 117) were found to exceed the acceptable overall percentage such as that shown in Figure 7.

**Figure 8:**
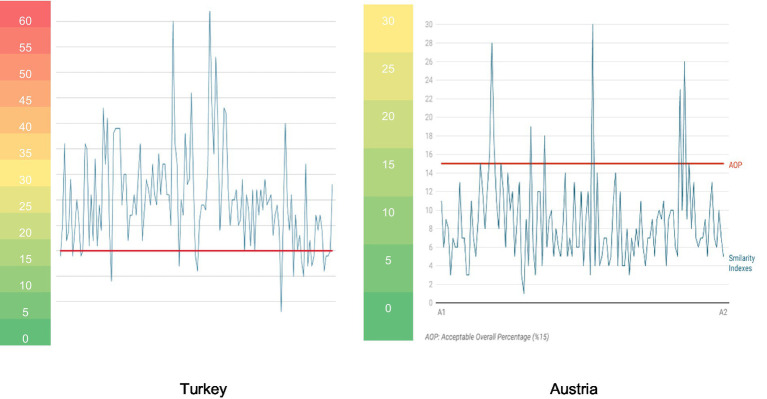
A comparison between similarity indexes of Turkey and Austria.

The mean similarity indexes for postgraduate theses written in the field of preschool education at Turkish universities was found to be 25.10 (std. dev. 9.85). In comparison, the mean similarity indexes for postgraduate theses prepared at Austrian universities was found to be 8.78 (std. dev. 4.91). Our analysis shows that the similarity indexes of most of the theses written in Turkey exceeded the acceptable overall percentage (%15) and some of exceeded the 60% level. In contrast, this finding does not apply to the Austrian sample. The comparative representation in Figure 8 reveals that the similarity indexes of the two countries differ dramatically from each other and shows the extent to which the critical level is breached.

### Findings related to research question 4

The Kruskal Wallis H-Test was employed to determine whether the similarity indexes of postgraduate theses (master’s and doctoral) written in the field of preschool education in Turkey varied significantly over the years. The results obtained are presented in Table 3.

**Table 3:** H-Test results of similarity indexes of postgraduate theses in Turkey grouped by year.

Year	*n*	Mean Rank	SD	$\chi^{2}$	*p*
2019	31	59.82	10	8.222	0.607
2018	26	70.87			
2017	15	79.77			
2016	7	85.29			
2015	14	75.96			
2014	4	74.00			
2013	11	68.86			
2012	5	58.20			
2011	8	60.19			
2010	8	47.69			
2009	5	56.70			

From the analysis results shown in Table 3 we can conclude that there was no significant difference between the mean ranks of the groups [H(10) = 8.222, *p* = 0.607].

### Findings related to research question 5

The Kruskal Wallis H-Test was employed to determine whether the similarity indexes of the postgraduate theses (master’s and doctoral) written in the field of pedagogy in Austria varied significantly over the years. The results obtained are presented in Table 4.

**Table 4:** H-Test results of similarity indexes of postgraduate theses in Austria grouped by year.

Year	*n*	Mean rank	SD	$\chi^{2}$	*p*
2018	16	55.84	10	10.838	0.370
2017	10	51.60			
2016	14	50.50			
2015	17	59.50			
2014	9	70.17			
2013	6	69.00			
2012	12	56.54			
2011	15	81.50			
2010	13	74.15			
2009	7	50.43			
2008	5	71.70			

We can see from the analysis results shown in Table 4 that there was no significant difference between the mean ranks of the groups [H(10) = 10.838, *p* = 0.370].

### Findings related to research question 6

The Mann Whitney U-Test was employed to determine whether the similarity indexes of the postgraduate theses (master’s and doctorate) written in the field of preschool education in Turkey and in the field of pedagogy in Austria differed from each other significantly. The results obtained are presented in Table 5.

**Table 5:** U-Test results of similarity indexes of postgraduate theses based grouped by country.

Country	*n*	Mean rank	Total ranks	U	*p*
Turkey	134	185.40	24843.50	817.500	0.000
Austria	124	69.09	8567.50		

The analysis results shown in Table 5 suggest that there was a significant difference between the mean ranks of the groups (U = 817.500, *p* = 0.000). Specifically, the mean rank of the similarity indexes of postgraduate theses written in Turkey (185.40) was substantially higher than the mean rank of the similarity indexes of postgraduate theses written in Austria (69.09).

The Mann Whitney U-Test was also used to test whether the similarity indexes of master’s and doctoral theses included in the data set differed significantly for each set across the countries. The analysis results obtained are presented in Table 6.

**Table 6:** U-Test results of similarity indexes of master’s and doctoral theses grouped by country.

Type of thesis	Country	*n*	Mean rank	Total ranks	U	*p*
Master’s	Turkey	111	152.44	16921.00	395.000	0.000
	Austria	100	54.45	5445.00		
Doctoral	Turkey	23	33.15	762.50	65.500	0.000
	Austria	24	15.23	365.50		

We can observe in the analysis results shown in Table 6 that there was a significant difference between the mean ranks of the groups (countries; U = 395.000, *p* = 0.000). Specifically, the mean rank of the similarity indexes of master’s theses written in Turkey (152.44) was substantially higher than the mean rank of the similarity indexes of master’s theses written in Austria (54.45).

In the same manner, the analysis of the doctoral theses indicates a significant difference between the mean ranks of the groups (U = 65.500, *p* = 0.000). Specifically, the mean rank of the similarity indexes of doctoral theses written in Turkey (33.15) was substantially higher than the mean rank of the similarity indexes of doctoral theses written in Austria (15.23).

### Findings related to research question 7

The Kruskal Wallis H-Test was employed to determine whether the similarity indexes of the postgraduate theses written in the field of preschool education in Turkey varied significantly over institutions. The results obtained are presented in Table 7.

**Table 7:** H-Test results of similarity indexes of postgraduate theses in Turkey grouped by institution.

College/university	*n*	Mean rank	SD	$\chi^{2}$	*p*
*blinded university	40	66.19	4	21.922	0.000
*blinded university	49	51.53			
*blinded university	20	92.78			
*blinded university	14	70.50			
*blinded university	11	93.64			

The analysis results shown in Table 7 suggest that there was a significant difference between the mean ranks of the groups [H(4) = 21.922, *p* = 0.000]. In terms of the mean ranks of the groups, the Middle East Technical University had the lowest similarity index (or produced more original postgraduate theses); it was followed by *blinded University, *blinded University, *blinded University and *blinded University.

A non-parametric multiple comparison test was performed after the Kruskal Wallis test to find out the groups between which the significant variation observed under the variable institution was most dominant. The result of this test showed that differences between the similarity indexes of postgraduate theses written at *blinded University and *blinded University and those written at *blinded University and *blinded University were statistically significant. Stated differently, we found that the similarity indexes of postgraduate theses written at *blinded University and *blinded University (92.78 and 93.64, respectively) were substantially higher than the similarity index of postgraduate theses written at the *blinded University (51.53).

### Findings related to research question 8

The Kruskal Wallis H-Test was employed to determine whether the similarity indexes of the postgraduate theses written in the field of pedagogy in Austria varied significantly over institutions. The results obtained are presented in Table 8.

**Table 8:** H-Test results of similarity indexes of postgraduate theses in Austria grouped by institution.

College/university	*n*	Mean rank	SD	$\chi^{2}$	*p*
*Blinded university	48	52.18	4	7.574	0.108
*Blinded university	44	72.50			
*Blinded university	14	66.21			
*Blinded university	14	62.93			
*Blinded university	4	61.88			

We can see from the analysis results shown in Table 8 that there was no significant difference between the mean ranks of the groups [H(4) = 7.574, *p* = 0.108].

### Findings related to research question 9

The Mann Whitney U-Test was used to determine whether the similarity index of master’s and doctoral theses written in Turkey differed from each other significantly. The results obtained are presented in Table 9.

**Table 9:** U-Test results of similarity indexes of postgraduate theses in Turkey grouped by thesis type.

Type of thesis	*n*	Mean rank	Total ranks	U	*p*
Master’s	111	74.27	8244.50	524.500	0.000
PhD	23	34.80	800.50		

We can observe from the analysis results shown in Table 9 that there was a significant difference between the mean ranks of the groups (U = 524.500, *p* = 0.000). Within this framework, in terms of mean ranks, the similarity indexes of master’s theses written in Turkey (74.27) are higher than the similarity indexes of the doctoral theses (34.80) from the same country.

### Findings related to research question 10

The Mann Whitney U-Test was used to determine whether the similarity indexes of master’s and doctoral theses written in Austria differed from each other significantly. The results obtained are presented in Table 10.

**Table 10:** U-Test results of similarity indexes of postgraduate theses in Austria grouped by thesis type.

Type of thesis	*n*	Mean rank	Total ranks	U	*p*
Master’s	100	60.68	6067.50	1017.500	0.247
PhD	24	70.10	1682.50		

The analysis results shown in Table 10 suggest that there was no significant difference between the mean ranks of the groups (U = 1017.500, *p* = 0.247).

## Discussion

In this study, the mean similarity index percentages for master’s and doctoral theses written in the field of preschool education in Turkey was calculated as 26.63 and 17.74, respectively. The mean similarity index for all postgraduate theses in Turkey was found to be 25.10 with a standard deviation of 9.85. These values are similar to the findings of the study conducted by Toprak (2017), which reviewed 600 postgraduate theses in the field of educational sciences in Turkey between 2007 and 2015 (although it shows a difference of 3.6%). Specifically, the Toprak study calculated a mean similarity index of 28.7 for postgraduate theses written in the field of educational sciences in Turkey. Toprak underscores that this similarity index points to the presence of a significant originality problem in reviewed theses and that, by the same token, these theses are studies that repeat each other in both theoretical and methodological dimensions. The same article points out that students have difficulty in writing original academic texts and that their works cannot go beyond repeating each other most of the time (Toprak, 2017). Similarly, Şen (2013) states that postgraduate theses in Turkey are not sufficiently original and that plagiarism is a steadily growing problem.

Several lines of evidence suggest that student cheating in general, and plagiarism in particular, are becoming more frequent and more widespread (Walker, 1998; Scanlon, 2003; Ballor, 2014; Ison, 2015; Tran et al., 2018; Laurent, 2020). Such impressive evidence comes from many countries, including the United States, the United Kingdom, Southern Africa, Finland, Canada and Vietnam “*embracing both undergraduate and postgraduate students and including public and private higher institutions of education, large and small”* (Park, 2003). A study conducted in Turkey with a study group of 308 prospective teachers reported that 91.5% of the participants engaged in plagiarism in their academic work (Avaroğulları and Ata, 2013). Another study conducted at one of the leading universities in Turkey reported that the students were not adequately informed about plagiarism and that they needed support in their academic writing (Köse and Arıkan, 2011).

Our findings show that doctoral theses written at Turkish universities have similarity indexes lower than those of master’s theses (U = 524.500, *p* = 0.000) at the same institutions. This may mean that more care is taken in doctoral studies to ensure originality (as compared to master’s studies). We also observed that, among the Turkish universities included in our sample, theses prepared at *blinded University, *blinded University, *blinded University, *blinded University and *blinded University have the lowest similarity indexes.

In the case of Austria, we find that postgraduate theses written in the field of pedagogy are quite original, the authors follow the rules of academic writing, and the similarity of the theses to existing works is low. The mean similarity indexes of master’s theses and doctoral theses in the field of pedagogy in Austria were calculated as 8.55 and 9.75, respectively. Our findings also indicate that the similarity indexes of theses written at different Austrian universities did not differ significantly from each other [H(4) = 7.574, *p* = 0.108]. Similarly, no significant differences were found between the similarity indexes of the doctoral and master’s theses written in Austria (U = 1017.500, *p* = 0.247). Based on these findings, one may conclude that Austrian universities maintain a certain standard in terms of quality of academic writing and that they do not differ from each other significantly.

We also observe that there is a very large difference between the similarity indexes of postgraduate theses written in Turkey and Austria. These results are consistent with the findings of intracultural studies which conclude that students from Turkey have a higher tendency to plagiarize compared to European students (Kayaoğlu et al., 2016). We found that the similarity indexes of only a small number of studies conducted in Turkey are below 15%, which we consider as the critical threshold. In contrast, the number of theses with similarity indexes that exceed this critical threshold is considerably low in Austria. The fact that similarity indexes of postgraduate theses written in Turkey are significantly higher than those of postgraduate theses written in Austria may mean that these two countries differ from each other in terms of originality and quality of academic writing in early childhood studies. Apart from these, it is noteworthy but also thought-provoking that 94% of the studies conducted in Austria employ qualitative research designs while only 33% of the studies conducted in Turkey are qualitative in nature.

## Conclusion

The aim of this study, based on the comparative investigation of postgraduate theses written in two countries in the field of pedagogy, was to attempt to give faculty, administrators, researchers, and decision-makers in the field of education policy a sense of questions about originality of academic research and quality of academic writing. Our findings suggest that 87% of the theses written in Turkey are dramatically similar to the available resources and many of the studies carry potential risks in terms of originality and plagiarism. The study results show that the majority of the theses prepared at Austrian universities (91%) have an acceptable similarity index. Our qualitative results suggest also that the 94% of the studies conducted in Austria are comprised of studies with a qualitative design and only 33% of the studies conducted in Turkey are comprised of qualitative studies. While observations according to Tavistock model, videography, video analysis, problem-centered interviews, narration interviews, content analysis, ethnography, Hermeneutics, Grounded Theory, biographical methods, and case studies were found to be widely used in studies conducted in Austria, most of the studies conducted in Turkey did not directly focus on children and instead addressed preschool teachers, prospective preschool teachers or parents. When we look at the general picture of the field of pedagogy in Turkey, we see that single-subject in-depth studies based on direct video analyses of preschool children or ethnographic and psychoanalytic observations are not are not as common as in Austria. This may be interpreted as the two countries having different paradigms and perspectives with respect to early childhood studies. We must also underscore that much of the research conducted in this field in Turkey is based on relational screening, surveys, or assessment instruments and that it consists of studies that are very similar to each other. These may all be signs of a need for a paradigm change in the area of pedagogy in Turkey.

On the one hand, it is necessary to emphasize that the present study revealed a number of interesting findings and insights in terms of originality, plagiarism, and different methodological approaches in the field of early childhood education in Austria and Turkey, and on the other hand, it must be pointed out that the intention in this article was not to conduct an analysis of the causes associated with academic plagiarism and diversity of scientific perspectives. It should be emphasized that future studies that explicitly use a triangulation framework based on surveys, interviews, text analysis, or other forms of data that are reflective of master and doctorate students’ practice and engagement should focus on the possible set of reasons for plagiarism. We suggest that ultimately, further qualitative studies can provide more considerable information and an accurate understanding on the growing literature on plagiarism in higher education, especially in developing countries, and the ways in which its incidence can be reduced.

The results from a qualitative study in Australia (Devlin and Gray, 2007) indicate a wide range of possible reasons for plagiarism, including: student’s understanding of plagiarism, poor academic skills, teaching and learning factors etc. The findings in the present study could indicate the need for further “plagiarism education” in research methodology courses in Turkish universities’ graduate curriculums. The integration of a plagiarism education into a research methodology course or a course on writing quality and academic ethics into institutions’ graduate curricula could be recommended to improve writing quality and increase the students’ academic awareness about what plagiarism includes (Eret and Gokmenoglu, 2010).

Furthermore, additional studies can be conducted to compare different countries (for example, to cover all OECD countries) across various disciplines and more comprehensive information can be obtained about the world of academic writing. Studies with larger samples with data from many countries and the publication of results in a manner similar to PISA reports will allow countries to have a sense of their level of academic originality and, more generally, contribute to academic progress and transformation to some extent.

## Data availability statement

The raw data supporting the conclusions of this article will be made available by the authors, without undue reservation.

## Author contributions

All authors contributed to the whole process through collecting the relevant literature, writing, and reviewing the manuscript. YP and DG planned the research design. KD, NP, EI, SS, and SÖ collected the data and previous literature. All authors contributed to the article and approved the submitted version.

## Conflict of interest

The authors declare that the research was conducted in the absence of any commercial or financial relationships that could be construed as a potential conflict of interest.

## Publisher’s note

All claims expressed in this article are solely those of the authors and do not necessarily represent those of their affiliated organizations, or those of the publisher, the editors and the reviewers. Any product that may be evaluated in this article, or claim that may be made by its manufacturer, is not guaranteed or endorsed by the publisher.

## Footnotes

^1^Ethnographic and psychoanalytical studies conducted directly with preschool children based on observation and videography techniques at the Educational Sciences Institutes of the University of *blinded and University of *blinded are particularly noteworthy.

^2^For example, “The Perceptions of Early Childhood Teachers towards Young Gifted Children” or “The Perceptions of Early Childhood Teachers towards Physical Design for Classroom Management” or “Review of Levels of Knowledge on Nutrition and Behaviors of Teachers” etc.

## References

[ref1] Altun D. Şendil Ç. Ö. Sahin İ. T. (2011). Investigating the national dissertation and thesis database in the field of early childhood education in Turkey. Procedia Soc. Behav. Sci. 12, 483–492. doi: 10.1016/j.sbspro.2011.02.060

[ref2] Avaroğulları M. Ata B. (2013). Sosyal Bilgiler Öğretmeni Adayları ve İntihal: Önbilgileri, İntihalin Yaygınlığı ve Başvurulan Yöntemler. Gaziosmanpaşa Bilimsel Araştırma Dergisi 4, 94–107.

[ref3] Aydinli E. Mathews J. (2021). Searching for larger status in global politics: internationalization of higher education in Turkey. J. Stud. Int. Educ. 25, 247–265. doi: 10.1177/1028315320932325

[ref4] Ballor J. J. (2014). Plagiarism in a digital age. J. Markets & Morality 17, 349–352.

[ref5] Bauer H. Mitterer K. (2014). “Der kindergarten als öffentliche institution [the preschool as a public institution]” in Handbuch Kindergartenleitung. Das ABC für Führungskräfte in der Elementarpädagogik [Handbook preschool management: The ABC for management in ECEC]. ed. Koch B. (Vienna: KiTa aktuell), 79–114.

[ref6] Behar L. B. (1977). The preschool behavior questionnaire. J. Abnorm. Child Psychol. 5, 265–275. doi: 10.1007/BF00913697 903521

[ref7] Bekman S. (2005). “Early childhood education in Turkey: an overview” in International perspectives on research in early childhood education. ed. Saracho O. N. Spodek B. (United States of America: Information Age Publishing Inc), 335–353.

[ref8] Bereswill M. Morgenroth C. Redman P. (2010). Alfred Lorenzer and the depth-hermeneutic method. Psychoanalysis, Culture & Society 15, 221–250. doi: 10.1057/pcs.2010.12

[ref9] Bowen G. A. (2009). Document analysis as a qualitative research method. Qual. Res. J. 9, 27–40. doi: 10.3316/QRJ0902027

[ref10] Christensen L. B. Johnson B. Turner L. A. (2011). Research Methods, Design, and Analysis. Boston, MA: Pearson Education, Inc.

[ref11] Cin F. M. Gümüş S. Weiss F. (2021). Women’s empowerment in the period of the rapid expansion of higher education in Turkey: developments and paradoxes of gender equality in the labour market. High. Educ. 81, 31–50. doi: 10.1007/s10734-020-00587-2

[ref12] Citron D. T. Ginsparg P. (2015). Patterns of text reuse in a scientific corpus. Proc. Natl. Acad. Sci. 112, 25–30. doi: 10.1073/pnas.1415135111, PMID: 25489072 PMC4291616

[ref13] Datler W. Hover-Reisner N. Trunkenpolz K. (2014). Observation according to the Tavistock model as a research tool: remarks on methodology, education and the training of researchers. Infant Observation. 17, 195–214. doi: 10.1080/13698036.2014.977558

[ref14] de Albuquerque Moreira A. M. Paul J. J. Bagnall N. (2019). “The contribution of comparative studies and cross-cultural approach to understanding higher education in the contemporary world” in Intercultural studies in higher education: Policy and practice (Cham: Springer International Publishing), 1–20.

[ref15] Denham S. A. (1986). Social cognition, prosocial behavior, and emotion in preschoolers: contextual validation. Child Dev. 57, 194–201. doi: 10.2307/1130651

[ref16] Devlin M. Gray K. (2007). In their own words: a qualitative study of the reasons Australian university students plagiarize. High. Educ. Res. Dev. 26, 181–198. doi: 10.1080/07294360701310805

[ref17] Dunn L. M. Dunn L. M. (1965). Peabody picture vocabulary test. Minneapolis: American Guidance Service, Inc.

[ref18] Eret E. Gokmenoglu T. (2010). Plagiarism in higher education: a case study with prospective academicians. Procedia Soc. Behav. Sci. 2, 3303–3307. doi: 10.1016/j.sbspro.2010.03.505

[ref19] Errami M. Hicks J. M. Fisher W. Trusty D. Wren J. D. Long T. C.. (2008). Déjà vu—a study of duplicate citations in Medline. Bioinformatics 24, 243–249. doi: 10.1093/bioinformatics/btm574, PMID: 18056062

[ref20] Frankenburg W. K. Dodds J. B. (1967). The Denver developmental screening test. J. Pediatr. 71, 181–191. doi: 10.1016/S0022-3476(67)80070-2 6029467

[ref21] George A. L. Bennett A. (2005). Case studies and theory development in the social sciences. Cambridge: MIT Press.

[ref22] Gür B. S. Çelik Z. Kurt T. Yurdakul S. (2017). Yükseköğretime bakış 2017: İzleme ve değerlendirme raporu [overview of higher education 2017: Monitoring and evaluation report]. Ankara: Eğitim-Bir-Sen Stratejik Araştırmalar Merkezi.

[ref23] Hammersley M. (1992). Some reflections on ethnography and validity. Int. J. Qual. Stud. Educ. 5, 195–203. doi: 10.1080/0951839920050301

[ref24] Hein J. Janke S. Rinas R. Daumiller M. Dresel M. Dickhäuser O. (2021). Higher education instructors’ usage of and learning from student evaluations of teaching–do achievement goals matter? Front. Psychol. 12:652093. doi: 10.3389/fpsyg.2021.652093, PMID: 34354628 PMC8329422

[ref25] Higher Education Information Management System. (2022). Yükseköğretim Bilgi Yönetim Sistemi. Retrieved October 08, 2022, from https://istatistik.yok.gov.tr

[ref26] Ireland, Christopher and English, John. (2011). Challenging and developing conceptions of plagiarism held by first year Accountancy undergraduates. Documentation. iParadigms Europe. Available at: http://eprints.hud.ac.uk/id/eprint/11242/

[ref27] Ison D. C. (2015). The influence of the internet on plagiarism among doctoral dissertations: an empirical study. J. Academic Ethics 13, 151–166. doi: 10.1007/s10805-015-9233-7

[ref28] Kalkan O. (2019). The transformation of higher education in Turkey between 2002–2018: An analysis of policies and politics of higher education (Master’s thesis, Middle East Technical University).

[ref29] Kandır A. Yazıcı E. (2016). Preschool education in Turkey. Current Adv. Educ. 15, 15–27.

[ref30] Kapci E. G. Guler D. (1999). Pre-school education in Turkey: policies and practices in their historical context. Early Child Dev. Care 156, 53–62. doi: 10.1080/0300443991560104

[ref31] Kayaoğlu M. N. Erbay Ş. Flitner C. Saltaş D. (2016). Examining students’ perceptions of plagiarism: a cross-cultural study at tertiary level. J. Furth. High. Educ. 40, 682–705. doi: 10.1080/0309877X.2015.1014320

[ref32] Koch B. Farquhar S. (2015). Breaking through the glass doors: men working in early childhood education and care with particular reference to research and experience in Austria and New Zealand. Eur. Early Child. Educ. Res. J. 23, 380–391. doi: 10.1080/1350293X.2015.1043812

[ref33] Köse Ö. Arıkan A. (2011). Reducing plagiarism by using online software: an experimental study, contemporary online language. Educ. J. 1, 122–129.

[ref34] Kvale S. (1995). The social construction of validity. Qual. Inq. 1, 19–40. doi: 10.1177/107780049500100103

[ref35] Kwiek M. Antonowicz D. (2013). “Academic work, working conditions and job satisfaction” in The work situation of the academic profession in Europe: Findings of a survey in twelve countries. eds. Teichler U. Höhle E. A. (Dordrecht: Springer), 37–54.

[ref36] Laurent M. R. (2020). Wikipedia, the free online medical encyclopedia anyone can plagiarize: time to address wiki-plagiarism. Publ. Res. Q. 36, 399–402. doi: 10.1007/s12109-020-09750-0

[ref37] Lessky F. Nairz E. Wurzer M. (2022). Social selectivity and gender-segregation across fields of study: comparative evidence from Austria. Int. J. Comp. Sociol. 63, 201–221. doi: 10.1177/00207152221099171

[ref38] Methlagl M. Taslimi N. J. Majcen J. (2022). Working as an ECE professional during Covid-19 in Austria: demands and resources profiles and their relations with exhaustion and work engagement. Professions and Professionalism 12:4642. doi: 10.7577/pp.4642

[ref39] Michałowska-Dutkiewicz A. Jóźwik K. Glendinning I. (2013). European Commission/Directorate General for Education and Culture, Impact of Policies for Plagiarism in Higher Education Across Europe. Plagiarism policies in Austria: Full report. Available at: http://plagiarism.cz/ippheae/files/D2-3-01%20AT%20IPPHEAE%20CU%20Survey%20austria.pdf

[ref40] Oktay A. (1999). Türkiye Cumhuriyetinin 75. yılında okul öncesi eğitim ve ilköğretim [preschool and primary school education in the 75th year of Turkish republic]. (pp. 137–161). Ankara: TÜBA.

[ref41] Özoǧlu M. Gür B. S. Gümüs S. (2016). Rapid expansion of higher education in Turkey: the challenges of recently established public universities (2006–2013). High Educ. Pol. 29, 21–39. doi: 10.1057/hep.2015.7

[ref42] Park C. (2003). In other (people’s) words: plagiarism by university students--literature and lessons. Assess. Eval. High. Educ. 28, 471–488. doi: 10.1080/02602930301677

[ref43] Pupovac V. (2021). The frequency of plagiarism identified by text-matching software in scientific articles: a systematic review and meta-analysis. Scientometrics 126, 8981–9003. doi: 10.1007/s11192-021-04140-5

[ref44] Reichertz J. Englert C. J. (2011). Einführung in die qualitative Videoanalyse. Wiesbaden: VS Verlag für Sozialwissenschaften.

[ref002] Scanlon P. M. (2003). Student online plagiarism: how do we respond?. College Teaching 51, 161–165.

[ref45] Şen Z. (2013). Türkiye’de Yüksek Lisans ve Doktora Eğitimi Kalitesinin İyileştirilmesi için Öneriler. Yükseköğretim ve Bilim Dergisi 1, 10–15.

[ref46] Smidt W. (2018). Early childhood education and care in Austria: challenges and education policies. Early Child Dev. Care 188, 624–633. doi: 10.1080/03004430.2017.1403431

[ref47] Smidt W. Embacher E. M. (2020). How do activity settings, preschool teachers’ activities, and children’s activities relate to the quality of children’s interactions in preschool? Findings from Austria. Eur. Early Child. Educ. Res. J. 28, 864–883. doi: 10.1080/1350293X.2020.1836586

[ref48] Strauss A. Corbin J. (1998). Basics of Qualitative Research: Techniques and Procedures for Developing Grounded Theory. Thousand Oaks. CA: Sage Publications, Inc.

[ref49] Sun Z. Errami M. Long T. Renard C. Choradia N. Garner H. (2010). Systematic characterizations of text similarity in full text biomedical publications. PLoS One 5, 1–6. doi: 10.1371/journal.pone.0012704, PMID: 20856807 PMC2939881

[ref50] Toprak Z. (2017). Türkiye’de Akademik Yazı: İntihal ve Özgünlük. Boğaziçi Üniversitesi Eğitim Dergisi 34, 1–12.

[ref51] Toprak Z. Yücel V. (2020). A peculiar practice of academic writing: epidemic writing in the Turkish graduate education. Cogent Educ. 7:1774098. doi: 10.1080/2331186X.2020.1774098

[ref52] Toran M. (2012). “Early childhood education in Turkey: a critical overview” in Neoliberal transformation of education in Turkey. eds. İnal K. Akkaymak G. (New York: Palgrave Macmillan)

[ref53] Tran U. T. Huynh T. Nguyen H. T. T. (2018). Academic integrity in higher education: the case of plagiarism of graduation reports by undergraduate seniors in Vietnam. J. Academic Ethics 16, 61–69. doi: 10.1007/s10805-017-9279-9

[ref001] Ural O. Ramazan M. O. (2007). “Türkiye’de okul öncesi eğitimin dünü ve bugünü. [Preschool education in Turkey: Past and present].” in Türkiye’de okul öncesi eğitim ve ilköğretim sistemi: temel sorunlar ve çözüm önerileri [Preschool and primary education in Turkey: Basic problems and solutions]. eds. Özdemir S., Bacanlı H. Sözer M. (Ankara: Türk Eğitim Derneği), 11–61.

[ref54] Walker J. (1998). Student plagiarism in universities: what are we doing about it? High. Educ. Res. Dev. 17, 89–106. doi: 10.1080/0729436980170105

[ref55] Willekens H. Scheiwe K. (2018). Looking Back–kindergarten and preschool in Europe since the late 18th century. Hildesheim: University; 2020.

[ref56] YÖK. (2019). Ulusal Tez Merkezi. Available at: https://tez.yok.gov.tr/UlusalTezMerkezi/Datafrom2008to2019 (Accessed October 8, 2022).

